# Neuroimaging-Guided Insights into the Molecular and Network Mechanisms of Chronic Pain and Neuromodulation

**DOI:** 10.3390/ijms27021080

**Published:** 2026-01-21

**Authors:** Chiahui Yen, Ming-Chang Chiang

**Affiliations:** 1 Department of International Business, Ming Chuan University, Taipei 111, Taiwan; chyen@mail.mcu.edu.tw; 2 Department of Life Science, College of Science and Engineering, Fu Jen Catholic University, New Taipei City 242, Taiwan

**Keywords:** chronic pain, neuroimaging, TMS, tES, neuromodulation, brain–computer interface, PET, MRS, neuroinflammation

## Abstract

Chronic pain is a pervasive and debilitating condition that affects millions of individuals worldwide. Unlike acute pain, which serves a protective physiological role, chronic pain persists beyond routine tissue healing and often arises without a discernible peripheral cause. Accumulating evidence indicates that chronic pain is not merely a symptom but a disorder of the central nervous system, underpinned by interacting molecular, neurochemical, and network-level alterations. Molecular neuroimaging using PET and MR spectroscopy has revealed dysregulated excitatory–inhibitory balance (glutamate/GABA), altered monoaminergic and opioidergic signaling, and neuroimmune activation (e.g., TSPO-indexed glial activation) in key pain-related regions such as the insula, anterior cingulate cortex, thalamus, and prefrontal cortex. Converging multimodal imaging—including functional MRI, diffusion MRI, and EEG/MEG—demonstrates aberrant activity and connectivity across the default mode, salience, and sensorimotor networks, alongside structural remodeling in cortical and subcortical circuits. Parallel advances in neuromodulation, including transcranial magnetic stimulation (TMS), transcranial electrical stimulation (tES), deep brain stimulation (DBS), and emerging biomarker-guided closed-loop approaches, provide tools to perturb these maladaptive circuits and to test mechanistic hypotheses in vivo. This review integrates neuroimaging findings with molecular and systems-level mechanistic insights into chronic pain and its modulation, highlighting how imaging markers can link biochemical signatures to neural dynamics and guide precision pain management and individualized therapeutic strategies.

## 1. Introduction

Chronic pain, affecting an estimated 20% of the global population, represents one of the most prevalent and challenging health problems worldwide [1]. Unlike acute pain, which serves as a protective biological signal for tissue damage, chronic pain persists beyond the normal healing period and reflects maladaptive reorganization of pain-processing systems [2,3]. It is increasingly recognized not merely as a symptom but as a distinct disease state characterized by persistent neurobiological alterations across molecular, cellular, and network levels of the central nervous system [4,5]. These maladaptive changes can profoundly affect quality of life, leading to physical disability, emotional distress, cognitive impairment, and social isolation [6].

Chronic pain is a multifactorial condition encompassing nociceptive, neuropathic, nociplastic, inflammatory, and psychosocial components [7,8]. Its pathophysiology involves complex interactions between peripheral and central sensitization, glial activation, neuroinflammation, neurotransmitter imbalance, and aberrant connectivity among pain-related brain networks [5,9]. Advances in neuroimaging have provided powerful tools to visualize and quantify these alterations, offering insights into the dynamic interplay between molecular signaling, neuronal circuitry, and behavior. fMRI, PET, and EEG have collectively revealed changes in brain regions such as the anterior cingulate cortex, insula, thalamus, prefrontal cortex, and somatosensory areas—regions integral to pain perception and modulation [10,11]. Nociplastic pain, a third mechanistic descriptor adopted by the IASP, refers to pain arising from altered nociception that is not fully explained by nociceptive or neuropathic mechanisms [12].

Despite significant progress in understanding pain mechanisms, effective management remains elusive. Conventional pharmacological interventions, including opioids, nonsteroidal anti-inflammatory drugs, antidepressants, and gabapentinoids (gabapentin/pregabalin), often yield limited efficacy and are associated with adverse effects and tolerance [13,14,15]. Similarly, physical therapy and surgical interventions provide only partial or temporary relief for many patients [16,17]. These limitations underscore the urgent need for novel therapeutic strategies that target the underlying neurobiological mechanisms of chronic pain rather than its symptomatic manifestations.

Therefore, there is a pressing need for practical, innovative approaches to alleviate chronic pain. Increasingly, chronic pain is understood through the lens of central nervous system plasticity, where brain structure and function are altered [18]. Neuroimaging [19,20,21] has played a crucial role in uncovering these mechanisms, guiding the development of brain-targeted interventions such as TMS and tES [22,23,24]. The purpose of this review is to explore the mechanisms underlying TMS and tES in the context of chronic pain treatment. By examining the related brain structures and networks, we aim to provide a comprehensive understanding of how these neuromodulation techniques exert their effects.

This article is intentionally a narrative review that synthesizes multimodal evidence linking molecular, cellular, and large-scale network mechanisms of chronic pain with neuroimaging biomarkers and neuromodulation. To guide literature inclusion, we prioritized (i) human neuroimaging studies (fMRI, PET, MRS, DTI, EEG/MEG) that explicitly quantified molecular or neurochemical markers (e.g., receptor binding potential, metabolite concentrations, glial activation) and/or network-level connectivity signatures; (ii) mechanistic or translational studies connecting these markers to pain phenotypes; (iii) neuromodulation studies that used imaging/physiological readouts to explain how stimulation perturbs molecular and network processes.

## 2. Molecular and Cellular Mechanisms of Chronic Pain Revealed by Neuroimaging

Chronic pain states are characterized by complex biochemical and molecular perturbations that alter neuronal excitability, synaptic transmission, and neuroimmune communication [5,25]. Neuroimaging PET, MRS, and neurochemical (advanced molecular tracers) studies have revealed widespread dysregulation of major neurotransmitter systems, including decreased GABAergic inhibition, increased glutamatergic excitability, and altered opioid receptor availability in key pain-related brain regions, such as the insula, anterior cingulate cortex (ACC), thalamus, and prefrontal cortex [26,27,28,29]. These alterations in the excitation–inhibition balance lead to cortical hyperexcitability and maladaptive synaptic plasticity, promoting the transition from acute nociceptive signaling to a chronic pain state.

### 2.1. Neurotransmitter Dysregulation

Neurotransmitter imbalance represents a fundamental mechanism in chronic pain pathophysiology [26,30]. PET studies using radio ligands for dopaminergic, serotonergic, GABAergic, and glutamatergic systems have revealed widespread dysregulation across pain-related networks [31,32]. Reduced binding potential of dopamine D2/D3 receptors in the striatum and prefrontal cortex has been observed in fibromyalgia and neuropathic pain, suggesting impaired reward and motivational processing [33,34]. Similarly, diminished GABAergic inhibition, reflected by reduced GABA levels on MRS and decreased GABAA receptor binding on PET, contributes to cortical hyperexcitability and central sensitization. Conversely, elevated glutamate and aspartate concentrations in the ACC and insula are associated with excitatory drive and pain persistence [35,36]. These molecular findings support the concept that chronic pain involves a shift toward an excitatory–inhibitory imbalance, disrupting cortical and subcortical homeostasis. From a “molecular neuroimaging” perspective, these neurotransmitter and receptor-level alterations can be indexed in vivo by MR spectroscopy measures of GABA and glutamatergic metabolites (Glx), and—where available—by receptor/transporter PET tracers, providing a direct bridge from synaptic chemistry to circuit-level phenotypes.

In addition to dopaminergic, opioidergic, glutamatergic, and GABAergic alterations, serotonergic signaling is a key modulator of chronic pain through both spinal and supraspinal mechanisms [37]. Descending 5-HT projections from the raphe nuclei can exert inhibitory or facilitatory control depending on receptor subtype and circuit context; for example, activation of 5-HT1A/5-HT7 pathways is generally associated with antinociceptive effects, whereas 5-HT2/5-HT3 signaling can promote nociceptive facilitation and central sensitization [38]. At the cortical and limbic level, serotonergic dysregulation interacts with affective-motivational processing and pain-related anxiety. Molecular imaging studies using PET tracers for the serotonin transporter (SERT) support a serotonergic contribution to pain processing [31]. At the same time, mechanistic reviews highlight how 5-HT-dependent synaptic plasticity in regions such as the ACC and insula can shape persistent pain states [10].

### 2.2. Neuroinflammation and Glial Activation

A hallmark of chronic pain is sustained activation of glial cells—microglia and astrocytes—that release proinflammatory cytokines (e.g., TNF-α, IL-1β, IL-6), chemokines, and reactive oxygen species, thereby amplifying nociceptive signaling [39,40]. Neuroimaging of glial activation has been made possible through PET tracers targeting the 18 kDa translocator protein (TSPO), a biomarker of microglial activation [41,42]. Elevated TSPO binding has been detected in the thalamus, insula, and somatosensory cortices of patients with neuropathic pain (NP) and complex regional pain syndrome, correlating with pain intensity and disease duration [43]. These findings provide direct in vivo evidence that neuroinflammation contributes to central sensitization and maladaptive plasticity. Complementary MRS studies have demonstrated increased myo-inositol, a marker of astrocytic activity, in similar brain regions, reinforcing the contribution of glial–neuronal interactions to chronic pain maintenance [44]. Importantly, TSPO PET and related inflammatory PET approaches can be viewed as “molecular imaging” readouts of glial activation states and neuroimmune tone, while cytokine-driven synaptic remodeling is expected to manifest as changes in functional connectivity and network dynamics.

Regarding TSPO, it is essential to note that TSPO is a mitochondrial outer-membrane protein involved in cholesterol transport, steroidogenesis, and mitochondrial homeostasis, and its expression is upregulated in activated microglia (and, context-dependently, astrocytes) during neuroinflammation [45,46]. Therefore, TSPO-PET is widely used as an in vivo proxy of neuroimmune activation rather than a direct readout of a single protective or harmful pathway. In chronic pain, elevated TSPO binding in regions such as the thalamus and insula has most consistently been interpreted as evidence of sustained glial activation associated with symptom severity [43,47]. However, we acknowledge that some TSPO-related mitochondrial functions may be compensatory in specific contexts.

### 2.3. Mitochondrial and Oxidative Stress Pathways

Molecular neuroimaging has also revealed disruptions in energy metabolism and oxidative stress regulation within pain-related circuits [32,48]. Altered N-acetylaspartate (NAA) levels—a surrogate marker of neuronal integrity—have been consistently reported in the prefrontal cortex, thalamus, and ACC in patients with fibromyalgia and chronic back pain [49]. Decreased NAA suggests mitochondrial dysfunction and neuronal loss, which may underpin cognitive and emotional disturbances frequently accompanying chronic pain [25,50]. Moreover, oxidative stress imaging using redox-sensitive PET tracers indicates an imbalance between reactive oxygen species production and antioxidant defense, linking cellular metabolic deficits to sustained nociceptive transmission. Mitochondrial and redox perturbations also have neuroimaging correlates: MRS-accessible metabolites (e.g., lactate and N-acetylaspartate) and metabolic PET measures can index bioenergetic stress, linking cellular energetics to macroscopic network vulnerability.

To clarify the link between mitochondrial dysfunction and the cognitive/emotional burden of chronic pain, reference [50] demonstrates that inflammation-induced mitochondrial and metabolic disturbances can drive the transition from acute to chronic pain by altering neuronal energy handling; such bioenergetic constraints can plausibly impact higher-order networks subserving cognition and affect. In parallel, reference [25] summarizes how persistent, LTP-like neuroplasticity and maladaptive circuit remodeling in prefrontal-limbic systems are associated with impaired executive control, mood dysregulation, and pain-related learning. Together, these works support our statement that mitochondrial/oxidative stress-related neuronal vulnerability and plasticity changes may contribute to cognitive and emotional disturbances that frequently accompany chronic pain [25,50].

### 2.4. Neuroplasticity and Synaptic Remodeling

Chronic pain involves long-term potentiation (LTP)-like mechanisms in pain pathways, resulting in synaptic remodeling and altered connectivity [25,33]. Molecular imaging of N-methyl-D-aspartate (NMDA) receptor activity using specific ligands has identified upregulated receptor availability in the insular and prefrontal cortices, aligning with electrophysiological evidence of cortical hyperexcitability [51,52]. These synaptic adaptations are thought to facilitate persistent pain memory traces and hinder the extinction of pain-related neural patterns. Neuroimaging studies have further shown that synaptic density markers, such as the synaptic vesicle glycoprotein 2A (SV2A) PET tracer, can quantify synaptic alterations associated with chronic pain, providing a potential biomarker for treatment monitoring. Increased NMDA receptor activity and SV2A PET binding suggest heightened synaptic turnover and persistent pain memory encoding. Such remodeling perpetuates aberrant signaling even in the absence of peripheral stimuli. Consistent with this, fMRI network reconfiguration can be framed as the systems-level output of molecular plasticity programs—BDNF–TrkB/CREB signaling, kinase–phosphatase balance, and AMPAR/NMDAR trafficking—that shift synaptic weights (LTP/LTD-like processes) across distributed pain-related circuits.

### 2.5. Integration of Molecular and Functional Imaging

Combining molecular neuroimaging with functional MRI has enabled researchers to correlate biochemical alterations with changes in network activity and connectivity. For instance, elevated glutamate levels in the ACC are linked with enhanced functional coupling between limbic and default mode networks, suggesting that neurotransmitter dysregulation drives network reorganization [53,54]. Similarly, increased TSPO expression in the thalamus and insula corresponds with hyperconnectivity in pain salience networks, highlighting the molecular basis of aberrant pain perception [55]. Such integrative approaches offer a systems-level perspective, connecting cellular and molecular disturbances to neural dynamics.

### 2.6. Ion Channels and Excitability Checkpoints

Beyond neurotransmitter availability, chronic pain is driven by excitability checkpoints governed by ion channels and transduction receptors along the axis [56,57]. Pain sensitization and ectopic firing are shaped by voltage-gated sodium (e.g., Nav1.7/1.8/1.9), calcium (e.g., Cav2.2), and potassium channel function, together with polymodal sensors such as TRP channels (TRPV1/TRPA1), ASICs, and purinergic receptors (e.g., P2X3) [58,59,60]. At the spinal level, activity-dependent changes in NMDA/AMPA receptor gating and phosphorylation amplify synaptic gain. At the same time, impaired inhibitory control (GABAergic/glycinergic signaling and chloride homeostasis) promotes disinhibition and facilitates central sensitization [61]. These molecular control points provide a direct mechanistic link to imaging-observed hyperexcitability phenotypes and offer targetable nodes for neuromodulation and pharmacotherapy.

### 2.7. Extracellular Vesicles and EV-miRNA Signaling

Extracellular vesicles (EVs), including exosomes released from neurons, glia, immune cells and stem cells [62], function as intercellular conveyors of proteins, lipids, and regulatory RNAs that can propagate or resolve pain-related signaling [63,64]. EV cargo—particularly miRNAs—can modulate neuroimmune pathways, synaptic plasticity, and mitochondrial responses in recipient cells. Because EVs can be sampled from biofluids, EV-miRNA signatures are promising candidates for minimally invasive biomarkers that may align with imaging-defined pain subtypes and predict neuromodulation response [65,66]. Therapeutically, engineered EVs represent a molecular delivery platform for anti-inflammatory or plasticity-normalizing cargo.

In summary, molecular neuroimaging provides critical insights into the neurochemical and inflammatory foundations of chronic pain (Table 1). By identifying in vivo biomarkers of neurotransmission, glial activation, and oxidative stress, these tools bridge the gap between microscopic cellular pathology and macroscopic brain network dysfunction. Understanding these mechanisms not only elucidates the pathogenesis of chronic pain but also informs the development of targeted neuromodulation strategies aimed at restoring neurochemical balance and synaptic homeostasis.

This table summarizes the principal molecular and cellular mechanisms implicated in the development and maintenance of chronic pain. The listed processes include peripheral and central sensitization, ion channel dysregulation, alterations in neurotransmitter and receptor systems, neuroinflammation, microglial and astrocytic activation, immune–neural interactions, changes in synaptic plasticity, and maladaptive intracellular signaling pathways (e.g., MAPK, NF-κB, CREB). Additional mechanisms such as epigenetic modifications, oxidative stress, mitochondrial dysfunction, altered neurotrophic factor signaling, and dysregulation of excitatory–inhibitory balance are included where relevant. Together, these processes contribute to heightened nociceptive transmission, amplification of network excitability, and the transition from acute to chronic pain states.

## 3. Network-Level Alterations in Chronic Pain: Insights from Functional and Structural Neuroimaging

Chronic pain is increasingly conceptualized as a disorder of large-scale brain networks rather than a dysfunction confined to isolated nociceptive regions [10,67]. Advances in neuroimaging, especially fMRI and diffusion tensor imaging (DTI), have transformed our understanding of how chronic pain alters both functional connectivity and structural integrity across distributed neural circuits [68]. These network-level changes reflect maladaptive neuroplasticity that reinforces pain perception, emotional dysregulation, and cognitive impairments characteristic of chronic pain syndromes [18,69]. Analyses further reveal that chronic pain disrupts the brain’s intrinsic architecture, reorganizing hubs, efficiency, and modular interactions essential for standard sensory, affective, and cognitive processing.

Importantly, network reconfiguration is not independent of molecular biology: persistent nociceptive input recruits glial and immune signaling, shifts neurotransmitter receptor/transport function, perturbs mitochondrial metabolism, and remodels synapses and myelin. These cellular programs can scale up to altered functional connectivity, oscillatory dynamics, and structural network topology. Accordingly, where available, we interpret network-level imaging findings alongside convergent molecular evidence (e.g., neuroinflammatory PET, MRS metabolites, and pharmacological or genetic links) to support a mechanistic, multiscale view of chronic pain.

### 3.1. Altered Functional Connectivity in Pain-Related Networks

Resting-state and task-based fMRI studies consistently demonstrate abnormal functional coupling within and between key pain-related networks, including the default mode network (DMN), salience network (SN), and sensorimotor network (SMN) [70]. The DMN—encompassing the medial prefrontal cortex (mPFC), posterior cingulate cortex (PCC), and precuneus—usually supports introspection and self-referential thought but exhibits hyperconnectivity and reduced deactivation during pain perception in chronic pain patients [71]. This persistent DMN activity is associated with rumination, catastrophizing, and the transition from acute to chronic pain states.

The salience network, anchored in the anterior insula and dorsal anterior cingulate cortex (dACC), is critical for detecting and prioritizing salient stimuli, including nociceptive inputs [72]. In chronic pain, increased SN activity and aberrant connectivity with the limbic system (e.g., amygdala, hippocampus) contribute to heightened pain salience and emotional amplification [73,74]. In contrast, the sensorimotor network demonstrates decreased connectivity between the primary somatosensory cortex (S1), secondary somatosensory cortex (S2), and supplementary motor areas, suggesting impaired sensory discrimination and motor control. Collectively, these network alterations underpin the clinical symptoms of hyperalgesia, allodynia, and pain persistence.

### 3.2. Thalamocortical and Limbic Dysregulation

The thalamus serves as a central relay integrating nociceptive signals from the periphery to cortical targets [75]. fMRI and PET studies reveal disrupted thalamocortical connectivity in neuropathic pain, characterized by increased coupling with limbic regions (insula, ACC) and decreased connectivity with prefrontal areas [68,76]. This imbalance shifts thalamic function from sensory relay toward affective processing, contributing to emotional distress and reduced pain inhibition. Structural MRI further identifies gray matter volume loss in the thalamus, insula, and prefrontal cortices, which correlates with pain duration and intensity [77,78]. Enhanced amygdala connectivity with both the mPFC and periaqueductal gray (PAG) underscores the emotional and autonomic components of chronic pain, consistent with its overlap with anxiety and depressive symptoms [3,79].

### 3.3. White Matter Microstructure and Structural Network Alterations

DTI studies provide complementary evidence for microstructural damage in white matter tracts supporting pain processing [80,81]. Reduced fractional anisotropy (FA) and increased mean diffusivity (MD) are frequently reported in the corpus callosum, cingulum bundle, internal capsule, and spinothalamic tracts, reflecting demyelination, axonal loss, or altered fiber coherence. These microstructural abnormalities correlate with both sensory and affective dimensions of pain, suggesting that chronic pain induces long-term remodeling of communication pathways between cortical and subcortical regions [10,76]. Longitudinal DTI analyses further indicate that successful pain relief, whether pharmacological or through neuromodulation, can partially reverse these white matter changes—supporting their functional significance and potential as imaging biomarkers of treatment response [82,83].

### 3.4. Dynamic Connectivity and Network Plasticity

Emerging approaches that analyze dynamic functional connectivity (dFC) have provided new insights into temporal fluctuations of network interactions [84]. Chronic pain patients exhibit reduced flexibility and decreased transitions between connectivity states, implying a loss of adaptive network dynamics [85]. Such rigidity in neural communication may underpin the persistence of pain perception and reduced responsiveness to sensory or emotional modulation. Importantly, neuromodulatory interventions such as TMS and transcranial direct current stimulation (tDCS) have been shown to normalize these dynamic patterns, restoring functional variability and enhancing inter-network communication [86,87].

In summary, chronic pain is characterized by widespread disruptions in the brain’s functional and structural connectome (Table 2). Aberrant connectivity within the DMN, SN, and SMN, together with thalamocortical and limbic dysregulation, underlies the sensory, affective, and cognitive dimensions of pain chronification. Integrating fMRI, DTI, and graph-theoretical analyses provides a comprehensive framework for understanding chronic pain as a disorder of network organization and plasticity. These insights lay the foundation for neuroimaging-guided neuromodulation strategies aimed at re-establishing standard connectivity and restoring adaptive network function.

By identifying functional and structural alterations at the network level in chronic pain through neuroimaging, these studies explored the roles of brain connectivity and specific regions (such as the prefrontal cortex and pre-defined pattern networks) in pain processing. This research employed a range of techniques, including fMRI, DTI, EEG/MEG, MRS, and PET, to elucidate the neurobiological basis of chronic pain. This overview highlights converging evidence that chronic pain is a disorder of distributed network dysfunction, rather than isolated regional abnormalities.

## 4. Neuroimaging-Guided Neuromodulation: Mechanisms and Therapeutic Implications

Advances in neuroimaging have profoundly shaped how neuromodulation interventions alter neural circuitry and restore neurochemical homeostasis to relieve chronic pain [8,88]. Noninvasive and invasive brain stimulation techniques—such as TMS, tDCS, transcranial alternating current stimulation (tACS), and deep brain stimulation (DBS)—are increasingly employed to restore disrupted network balance and normalize aberrant activity within pain-related circuits [22,89]. These studies highlight the regulation of cortical and subcortical regions closely associated with pain perception, emotion regulation, and cognitive control. In particular, neuroimaging demonstrates that neuromodulatory interventions normalize thalamocortical dynamics, improve prefrontal inhibitory function, and enhance endogenous analgesic mechanisms within descending pain pathways [33,76]. Integrating multimodal neuroimaging with neuromodulation not only enhances mechanistic understanding but also supports precision-guided pain management, tailoring interventions to the individual’s neural architecture and pathophysiology.

### 4.1. TMS

TMS delivers brief magnetic pulses through the scalp to induce electric currents in cortical tissue, modulating neuronal excitability and synaptic plasticity. Repetitive TMS (rTMS) applied to the primary motor cortex (M1) or dorsolateral prefrontal cortex (DLPFC) can engage distributed analgesic circuits, including thalamus, anterior cingulate cortex (ACC), insula, and brainstem descending control (PAG–RVM), as demonstrated by fMRI and PET. At the molecular level, imaging and physiological studies support effects on endogenous opioid release, monoaminergic signaling, and excitatory–inhibitory rebalancing (glutamate/GABA), consistent with improvements in both sensory-discriminative and affective-motivational pain dimensions. Mechanistically, rTMS can bias cortical circuits toward LTP-like potentiation (typically high-frequency protocols) or LTD-like depression (typically low-frequency protocols) through NMDA receptor-dependent Ca2+ signaling and BDNF/TrkB-mediated synaptic stabilization, thereby helping to normalize maladaptive plasticity and restore more physiological patterns of network dynamics [76,90,91,92,93,94,95,96,97].

Mechanistically, rTMS protocols are often discussed in terms of LTP- and LTD-like plasticity because patterned stimulation can bias NMDA receptor-dependent Ca2+ signaling and downstream kinase/phosphatase balance, thereby modulating AMPA receptor trafficking and synaptic gain [98,99]. Although the net direction of after-effects is state-dependent, high-frequency rTMS and intermittent theta-burst stimulation (iTBS) more commonly induce facilitatory, LTP-like signatures [100,101]. In contrast, low-frequency rTMS and continuous TBS (cTBS) tend to produce inhibitory, LTD-like signatures in common human proxies of corticospinal and cortical excitability.

At the molecular level, these plasticity-like effects are supported by activity-dependent signaling cascades including CaMKII/ERK pathways, CREB-related transcription, and neurotrophin modulation (notably BDNF–TrkB), which together help stabilize longer-lasting changes consistent with late-phase plasticity [102,103]. In the context of chronic pain, where excitatory–inhibitory imbalance and maladaptive network “set points” can constrain physiological learning rules, rTMS may therefore be conceptualized as restoring the dynamic range of synaptic plasticity (metaplasticity) rather than simply increasing or decreasing excitability, enabling network reconfiguration toward more adaptive states and improving engagement of descending inhibitory control.

### 4.2. tDCS

tDCS applies weak direct currents (typically 1–2 mA) through scalp electrodes to polarize neuronal membranes and shift resting potential, producing polarity-dependent changes in cortical excitability. Neuroimaging studies indicate that anodal DCS targeting M1 or the DLPFC can downregulate hyperactivity in salience/limbic regions while strengthening functional coupling with descending inhibitory pathways. MRS evidence further suggests that tDCS can increase cortical GABA and reduce glutamate in targeted areas, providing a neurochemical substrate for restoring excitatory–inhibitory balance. PET studies also report modulation of mesocorticolimbic dopamine and regional blood flow, aligning with effects on motivation, affect, and cognitive control that often co-vary with pain relief [104,105,106,107,108,109,110,111,112,113,114].

### 4.3. tACS

tACS delivers oscillatory currents that entrain neuronal firing at specific frequencies, enabling targeted modulation of neural synchrony and inter-regional communication. Because chronic pain is associated with thalamocortical dysrhythmia and altered oscillatory balance (e.g., increased theta and reduced alpha/beta), frequency-specific tACS can be used to test causal links between pathological rhythms and pain phenotypes. EEG/MEG and concurrent fMRI studies suggest that alpha- or gamma-band tACS over sensorimotor and frontoparietal regions can reshape coherence within pain-relevant networks and modulate inhibitory interneuron function, thereby influencing excitability, attentional allocation, and salience attribution [115,116,117,118,119,120,121,122,123].

### 4.4. DBS

DBS involves the surgical implantation of electrodes into specific subcortical targets to deliver continuous or patterned electrical stimulation that modulates aberrant neuronal firing. In refractory neuropathic pain, commonly targeted regions include the periaqueductal/periventricular gray (PAG/PVG), sensory thalamus, and anterior cingulate cortex (ACC). Neuroimaging supports DBS-induced normalization of activity within pain-processing regions (insula, somatosensory cortices, prefrontal cortex) together with engagement of brainstem-descending inhibitory pathways and limbic-prefrontal circuits that shape the affective component of pain. Diffusion tractography and connectivity mapping are increasingly used to personalize target selection and parameter tuning by identifying patient-specific structural pathways that mediate clinical benefits [22,68,124,125,126,127,128,129,130].

### 4.5. Brain–Computer Interfaces and Closed-Loop Neuromodulation

Beyond open-loop stimulation, brain–computer interfaces (BCIs) and related neurofeedback systems are emerging as closed-loop approaches that use neuroimaging or electrophysiological biomarkers to deliver sensory or neuromodulatory interventions adaptively [131,132]. In chronic pain, EEG-informed BCI paradigms can target pathological oscillatory signatures (e.g., frontal theta or sensorimotor rhythms) and provide real-time feedback to reshape network dynamics. For example, a pilot theta-controlled vibrotactile neurofeedback BCI was reported to reinforce frontal theta activity in patients with chronic pain. It was associated with reductions in pain intensity, illustrating the feasibility of biomarker-guided, non-pharmacological modulation [133]. Simultaneously, implantable BCI platforms are rapidly advancing, including Neuralink’s brain–computer interfaces, with implications for medical innovation [134,135]. However, most are currently developed for motor restoration; their long-term potential for adaptive pain modulation will depend on the identification of robust neural pain signatures and the validation of closed-loop control laws. Together, BCIs extend the neuroimaging-guided neuromodulation framework toward individualized, state-dependent interventions that explicitly couple bi-omarkers to stimulation or sensory feedback.

### 4.6. Neurobiological Substrates of Neuromodulation Analgesia: Neurotransmitter and Neuroimmune Mechanisms

Beyond macroscopic circuit reconfiguration, convergent molecular mechanisms help explain why stimulation-based interventions can produce sustained analgesia. Across modalities, evidence supports changes in neurotransmitter tone (including synthesis and release), astrocyte-mediated glutamate clearance, and glia–immune signaling that together shift excitatory–inhibitory balance and plasticity thresholds.

Neurotransmitter circuits and inhibitory control: Noninvasive cortical stimulation can bias local neurochemistry, as measured by MRS. For example, anodal tDCS over the motor cortex has been associated with reductions in local GABA concentration and changes in glutamatergic measures (Glx), consistent with altered cortical excitability and plasticity [110,136]. At the systems level, rTMS/tDCS may also influence descending modulatory pathways (serotonergic, noradrenergic, and opioid) that gate spinal nociceptive transmission by modulating transmitter availability, receptor signaling, and synaptic release probability [137,138].

Glutamate reuptake and astrocyte function: A key molecular determinant of excitotoxic drive is the efficiency of glutamate uptake by astrocytic transporters (EAAT1/GLAST and EAAT2/GLT-1) and subsequent conversion to glutamine via glutamine synthetase [139]. Preclinical evidence suggests that tDCS can enhance astrocytic glutamate uptake capacity and facilitate glutamine synthesis, thereby reducing extracellular glutamate and excitatory stress [140,141]. This astrocyte-centered axis offers a natural bridge to neuroimaging markers of excitatory–inhibitory balance (MRS) and network dynamics (functional connectivity).

Microglial activation and cytokine signaling: Multiple neuromodulation modalities appear to engage anti-inflammatory pathways. In animal and translational studies, rTMS has been reported to reduce microglial/astrocytic activation markers (e.g., Iba1, GFAP) and to decrease pro-inflammatory cytokines (e.g., IL-1β, IL-6, TNF-α), while increasing anti-inflammatory mediators [142,143]. Mechanistic studies also indicate that microglial cytokine release can modulate stimulation-induced plasticity, highlighting a biologically plausible link among neuromodulation, neuroimmune tone, and long-term synaptic remodeling [33,144]. In invasive approaches, spinal cord stimulation (SCS) has been connected to glia-mediated processes, and microglial states may influence the net inhibitory effect of GABAergic signaling during SCS [145,146]. In parallel, vagus nerve stimulation (VNS) has well-established anti-inflammatory effects through neuroimmune regulation (e.g., the cholinergic anti-inflammatory pathway), providing a complementary mechanism for reducing peripheral and central inflammatory signaling relevant to chronic pain [147,148,149].

Taken together, these molecular and cellular mechanisms—neurotransmitter tone (glutamate/GABA and monoamines), astrocytic glutamate clearance, and microglia-driven cytokine signaling—provide testable “target engagement” hypotheses for neuroimaging-guided neuromodulation. They motivate multimodal study designs that combine network readouts (fMRI/DTI), neurochemical measures (MRS), and molecular inflammation markers (e.g., TSPO PET or peripheral biomarkers) to refine patient stratification and predict treatment response.

In summary, neuroimaging-guided neuromodulation elucidates both the mechanistic foundations and therapeutic potential of brain stimulation in chronic pain (Table 3). By revealing how interventions such as TMS, tDCS, tACS, DBS, and emerging closed-loop BCI/neurofeedback systems modulate neurochemical balance, neuroimmune activation, network connectivity, and oscillatory dynamics, imaging provides a blueprint for precision-targeted, adaptive treatment paradigms. The convergence of molecular, functional, and structural imaging with neuromodulatory technologies represents a pathway toward mechanism-based pain management that aims to restore network function rather than provide symptomatic relief alone. This table summarizes the primary neuromodulation techniques applied to modulate pain-related neural circuits, including TMS, tDCS, tACS, DBS, and emerging BCI/neurofeedback approaches.

### 4.7. Limitations and Future Directions

Several limitations should be considered when interpreting neuroimaging biomarkers and neuromodulation effects in chronic pain. First, chronic pain populations are heterogeneous with respect to etiology (nociceptive, neuropathic, nociplastic), comorbid mood disorders, and medication use, all of which can influence molecular and network readouts. Second, many imaging markers (e.g., TSPO-PET binding, resting-state connectivity, oscillatory power) are indirect and can vary across scanners, preprocessing pipelines, tracer properties, and analysis choices, limiting cross-study comparability. Third, most studies remain cross-sectional and cannot fully resolve causality between molecular alterations, network dysfunction, and symptom persistence. Future work that combines longitudinal, multimodal imaging with individualized electric-field modeling and closed-loop stimulation will be critical for establishing causal mechanisms and optimizing precision pain interventions.

## 5. Conclusions

**Figure 1:**
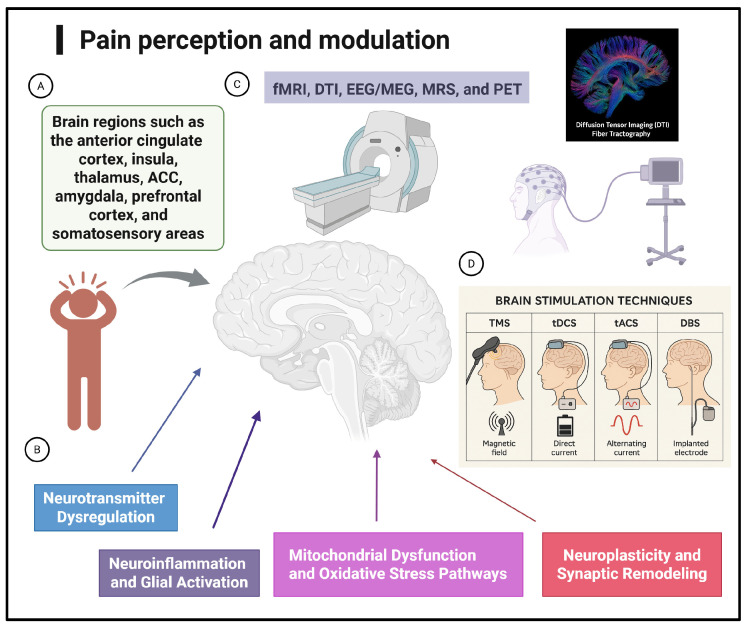
Pain perception and modulation: brain circuits, molecular pathways, neuroimaging markers, and neuromodulation interventions. This infographic illustrates the multilevel framework of pain processing and modulation. (**A**) Key brain regions involved in pain perception include the ACC, insula, thalamus, amygdala, prefrontal cortex, and somatosensory areas. (**B**) Central molecular and cellular mechanisms underlying chronic pain, including neurotransmitter dysregulation, neuroinflammation and glial activation, mitochondrial dysfunction, oxidative stress pathways, and maladaptive neuroplasticity. (**C**) Multimodal neuroimaging approaches, including fMRI, DTI, EEG/MEG, MRS, and PET, are used to characterize structural, functional, and metabolic alterations in chronic pain. (**D**) Noninvasive and invasive neuromodulation techniques are used to modulate pain-related circuits, including TMS, tDCS, tACS, and DBS. In this diagram, arrows indicate which factors in the pain network influence the brain circuits that generate and regulate pain perception. The figure was created with BioRender. Created in BioRender. Chiang, M. (2026) https://BioRender.com/ht5v3kb (accessed on 13 January 2026).

Neuroimaging has illuminated the pathophysiology of chronic pain at both the molecular and systems levels, revealing how alterations in neurotransmission, network connectivity, and neuroinflammation contribute to pain chronification. As neuromodulation techniques such as TMS and tES continue to evolve, imaging biomarkers will play a pivotal role in personalizing treatment, monitoring therapeutic response, and informing mechanism-based innovations in pain neuroscience. The diagram illustrating chronic pain mechanisms highlights the interactions between molecular, cellular, and network-level processes (Figure 1). Figure 1 shows the impact of neurotransmitter imbalance, neuroinflammation, glial cell activation, and changes in brain connectivity. Ultimately, integrating multimodal neuroimaging and targeted neuromodulation represents a transformative approach to chronic pain management—shifting from symptom relief to precision-guided restoration of brain network function.

To further advance mechanism-based translation, future studies should integrate molecular imaging (e.g., receptor and neuroinflammatory PET, MRS metabolites) with cell-type-resolved molecular profiling (transcriptomics, proteomics, epigenetic and extracellular vesicle signatures) and longitudinal network analyses. Such multiscale biomarkers can support patient stratification, quantify target engagement during neuromodulation, and enable adaptive, closed-loop stimulation strategies grounded in measurable molecular pathways rather than symptoms alone.

## Figures and Tables

**Table 1:** Molecular and Cellular Mechanisms of Chronic Pain.

Study	Effects	Reference
Huang D et al. (2025)	Chronic pain through the glutamate–GABA “tug-of-war” clarifies how molecular and glial mechanisms scale to network dysfunction and behavior, and points to precision, cortex-focused interventions that restore excitatory–inhibitory (E/I) balance.	[36]
Chaudhari A et al. (2025)	ICAM-1 (intercellular adhesion molecule-1) is a central regulator of neuroinflammation driving NP. Targeting ICAM-1–mediated neuroimmune crosstalk offers a pathway to disease-modifying therapies in NeP.	[39]
Singh P et al. (2022)	TSPO (18 kDa translocator protein) is a mitochondrial membrane protein (located at the outside-inside contact site) involved in steroid production, cholesterol transport, apoptosis, mitochondrial respiration, and cell proliferation. TSPO has been reported to be upregulated in neuroinflammatory states, brain injury, and systemic inflammation, thus serving as an essential target for in vivo imaging of neuroinflammatory conditions (including chronic pain and neuropathic pain).	[41]
Weerasekera A et al. (2024)	Thalamus MRI metabolites (decreased NAA, decreased Cho, increased mIns level) are potential cross-disease biomarkers for chronic musculoskeletal pain, including chronic lower back pain and knee osteoarthritis.	[49]
Willemen H et al. (2023)	The redox pathway of the target neuronal mitochondrial protein (ATPSc-KMT) may prevent or reverse chronic inflammatory pain, suggesting that mitochondrial dysfunction is a modifiable driver in the chronicity process.	[50]
Wey HY et al. (2014)	Simultaneous fMRI-PET can link neurochemistry and hemodynamics in vivo, providing a framework for identifying specific components of neurotransmitters and mapping how the dynamics of endogenous opioids affect neural network activation and neurovascular coupling.	[28]

**Table 2:** Network-Level Functional and Structural Alterations in Chronic Pain Identified by Neuroimaging.

Study	Effects	Reference
Fiúza-Fernandes J et al., 2025	Resting-state fMRI studies of chronic pain were analyzed to compare patients with chronic pain and healthy controls. Chronic pain is characterized by maladaptive remodeling of intrinsic brain networks, particularly involving DMN nodes (mPFC, precuneus), insula, and descending pain control pathways.	[70]
Kucyi A et al., 2014	Resting-state fMRI was used to investigate the relationship between the connectivity of the predefined pattern network (DMN) and pain-related rumination in individuals with chronic pain, with a focus on the medial prefrontal cortex (mPFC). In the patient group, higher pain rumination scores were associated with stronger connectivity between the mPFC and the posterior cingulate cortex/precuneus, posterior cingulate cortex, medial thalamus, and periaqueductal/perivectric gray matter. These results suggest that excessive communication within the DMN and between the DMN and descending regulatory structures is a neural mechanism supporting maladaptive rumination in chronic pain.	[71]
Ong WY et al., 2019	The multifaceted role of the PFC in pain management is highlighted, emphasizing its rich connectivity and plasticity in both acute and chronic pain. The PFC participates in mediating the dynamics and treatability of pain dysfunction by projecting to other cortical areas, the hippocampus, thalamus, amygdala, basal ganglia, and especially the PAG.	[76]
Lam J et al., 2024	An exploratory case–control structural and diffusion-weighted MRI study was conducted to investigate the relationship between brain morphology and pain distribution in patients with chronic overlapping pain disorders. Primary outcome measures included subcortical volume, cortical thickness, white matter microstructure, and whole-brain gray matter intensity. Compared to chronic pain patients and controls, patients had reduced right thalamic volume, and this smaller right thalamic volume was associated with higher pain intensity and more severe pain-related functional impairment. Compared to controls, the patient group also had reduced right prefrontal cortical thickness, and this thinner cortex was associated with higher pain intensity.	[78]
Zhang Y et al., 2020	An automated DTI fiber tractography method was developed to reconstruct nine key brainstem fiber pathways associated with pain modulation and to test their correlation with pain severity. This study demonstrates the feasibility and physiological relevance of automated DTI fiber tractography for brainstem circuits involved in pain modulation.	[81]
Yang S et al., 2021	The application of DTT in the diagnosis and understanding of NP focuses on the relationship between tract-level damage and the pathophysiology of NP. DTT can visualize and quantify nerve tract damage at the microscopic level, serving as an auxiliary diagnostic and mechanistic tool for NP.	[83]

**Table 3:** Neuromodulation Modalities (TMS, tDCS, tACS, DBS, and BCI/neurofeedback) for Regulating Pain-Related Neural Circuits.

Study	Effects	Reference
Kong Q et al., 2024	Non-invasive brain stimulation (NIBS)—including TMS, tDCS, and related therapies—is increasingly being used to treat chronic pain. The bilateral M1, supplementary motor area (SMA), precentral tegmentum, and temporoparietal junction (TPJ) are promising target areas, while EEG mapping can help improve their clinical application value.	[22]
Galanis C et al., 2025	Existing rTMS protocols show that 10 Hz repetitive magnetic stimulation drives BDNF/TrkB-dependent, STDP-like potentiation of excitatory synapses through cooperative pre- and postsynaptic activation, offering a mechanistic bridge between rTMS parameters and synaptic-level plasticity that can inform future experimental and clinical neuromodulation strategies.	[91]
Nardone R et al., 2017	NP following spinal cord injury (SCI) is often severe and complex to treat. Studies have shown that 10 Hz rTMS of the motor cortex/DLPFC can alleviate NP in SCI patients, at least in the short-to-medium term.	[92]
Ho K-A et al., 2016	tDCS delivers weak electrical currents to modulate cortical excitability. Its effects are strongly influenced by current density, which is determined by the following: Current intensity (e.g., 1 mA vs. 2 mA) and electrode size. Electrode size—not current intensity—is the primary determinant of tDCS-induced motor cortical excitability, with larger (35 cm^2^) electrodes producing more substantial and cumulative excitatory effects, highlighting the importance of careful electrode placement and field modeling in designing effective tDCS protocols.	[109]
Mondino M et al., 2019	Even a single low-intensity (1 mA) session of tDCS or tACS over bilateral DLPFC can rapidly and measurably increase fronto-parietal resting-state connectivity, demonstrating that transcranial current stimulation modulates large-scale networks in vivo and underscoring the utility of concurrent tCS–fMRI approaches for mechanistic and translational work.	[107]
Ta Dinh S et al., 2019	Chronic pain is highly prevalent and disabling, and current treatments are often inadequate. Chronic pain is characterized not by simple resting EEG power changes, but by increased theta and gamma synchrony and network reorganization in frontal regions, suggesting abnormal frontal connectivity as both a mechanical feature of chronic pain and a promising target for neuromodulation or neurofeedback.	[115]
Cirillo J et al., 2025	Applying gamma-frequency tACS to the M1 reduces GABA-mediated intracortical inhibition, potentially enhancing cortical excitability. Applying gamma-frequency tACS to M1 selectively modulates GABA-A-mediated inhibition in a frequency- and intensity-dependent manner—most significantly at 75 Hz and 1.5–2.0 mA—but does not reliably alter motor excitability. This underscores the complex necessity of gamma-band neuromodulation and the need for exploratory frequencies across different age groups.	[122]
Liao WY et al., 2025	Intermittent theta rhythmic stimulation (iTBS) of the M1 induces LTP-like plasticity, but its intensity varies among individuals. γ-tACS at 70 Hz enhances this plasticity, and recent studies suggest that this enhancement is frequency-specific, primarily concentrated in the mid-γ frequency range (60–90 Hz). Simultaneous γ-tACS and iTBS stimulation of M1 selectively enhances LTP-like plasticity, with the most substantial effect at 90 Hz. Furthermore, short-term inhibition (SICI) decreases across γ frequencies—highlighting the importance of frequency-specific optimization of γ-tACS to enhance motor cortical plasticity.	[123]
Fontaine D et al., 2025	DBS for chronic pain primarily targets the sensory thalamus or periaqueductal gray matter, but its efficacy varies and is often unsatisfactory. In recent years, given the ACC’s role in the emotional dimension of pain, this area has been proposed as a novel target. DBS treatment of the anterior cingulate cortex, alone or in combination with thalamic DBS, is feasible and safe in treating chronic refractory NP and has been shown to improve patients’ quality of life. This supports further development of ACC-centered neural circuit intervention techniques for the treatment of intractable pain.	[126]
Demarest P et al., 2024	EEG frontal theta modulation drives vibrotactile feedback; neurofeedback-BCI regulation of pain-related networks. Longitudinal open-label pilot (n = 6, chronic upper-extremity pain): Theta-gated neuromodulation is hypothesized to engage state-dependent plasticity and top-down control, reinforcing a pain relief–associated frontal theta state and potentially reweighting salience/affective network dynamics.	[133]

## Data Availability

No new data were created or analyzed in this study. Data sharing is not applicable to this article.
